# Protocol for an automated, pragmatic, embedded, adaptive randomised controlled trial: behavioural economics-informed mobile phone-based reminder messages to improve clinic attendance in a Botswanan school-based vision screening programme

**DOI:** 10.1186/s13063-022-06519-y

**Published:** 2022-08-15

**Authors:** Luke N Allen, Bakgaki Ratshaa, David Macleod, Nigel Bolster, Matthew Burton, Min Kim, Andrew Bastawrous, Ari Ho-Foster, Hannah Chroston, Oathokwa Nkomazana

**Affiliations:** 1 LSHTM, Keppel St, London, WC1E 7HT UK; 2 University of Botswana, Gaborone, Botswana; 3 Peek Vision, Berkhamsted, UK

**Keywords:** Behavioural economics, Reminder messages, mHealth, Health services research, Adaptive RCT

## Abstract

**Background:**

Clinic non-attendance rates are high across the African continent. Emerging evidence suggests that phone-based reminder messages could make a small but important contribution to reducing non-attendance. We will use behavioural economics principles to develop an SMS and voice reminder message to improve attendance rates in a school-based eye screening programme in Botswana.

**Methods:**

We will test a new theory-informed SMS and voice reminder message in a national school-based eye screening programme in Botswana. The control will be the standard SMS message used to remind parents/guardians to bring their child for ophthalmic assessment. All messages will be sent twice. The primary outcome is attendance for ophthalmic assessment. We will use an automated adaptive approach, starting with a 1:1 allocation ratio.

**Discussion:**

As far as we are aware, only one other study has used behavioural economics to inform the development of reminder messages to be deployed in an African healthcare setting. Our study will use an adaptive trial design, embedded in a national screening programme. Our approach can be used to trial other forms of reminder message in the future.

**Trial registration:**

ISRCTN 96528723. Registered on 5 January 2022.

**Supplementary Information:**

The online version contains supplementary material available at 10.1186/s13063-022-06519-y.

## Administrative information

Note: the numbers in curly brackets in this protocol refer to SPIRIT checklist item numbers. The order of the items has been modified to group similar items (see http://www.equator-network.org/reporting-guidelines/spirit-2013-statement-defining-standard-protocol-items-for-clinical-trials/).

**Table:** 

Title {1}	Protocol for an automated, pragmatic, embedded, adaptive randomised controlled trial: behavioural economics-informed mobile phone-based reminder messages to improve clinic attendance in a Botswanan schools-based vision screening programme
Trial registration {2a and 2b}.	ISRCTN96528723
Protocol version {3}	Version 1. January 2022.
Funding {4}	This work was supported by the National Institute for Health Research (NIHR) (using the UK’s Official Development Assistance (ODA) Funding) and Wellcome [grant reference 215633/Z/19/Z] under the NIHR-Wellcome Partnership for Global Health Research.
Author details {5a}	Dr Luke N Allen, luke.allen@lshtm.ac.uk, LSHTM Bakgaki Ratshaa, bakgaki@gmail.com, University of Botswana Dr David Macleod, david.macleod@lshtm.ac.uk, LSHTM Dr Nigel Bolster, nigel@peekvision.org, Peek Vision and LSHTM Prof Matthew Burton, matthew.burton@lshtm.ac.uk, LSHTM Min Kim, min.kim@lshtm.ac.uk, LSHTM Prof Andrew Bastawrous, andrew.bastawrous@lshtm.ac.uk, LSHTM and Peek Vision Mr Ari Ho-Foster, hofostera@ub.ac.bw, University of Botswana Mrs Hannah Chroston, Hannah.Chroston@lshtm.ac.uk, LSHTM Prof Oathokwa Nkomazana, nkomazanao@UB.AC.BW, University of Botswana
Name and contact information for the trial sponsor {5b}	**London School of Hygiene & Tropical Medicine** For further information regarding the sponsorship conditions, please contact the Research Governance and Integrity Office: London School of Hygiene & Tropical Medicine Keppel Street London WC1E 7HT Tel: +44 207 927 2626 Email: RGIO@lshtm.ac.uk
Role of sponsor {5c}	Delegated responsibilities will be assigned locally. The study funders will not have any role in- or ultimate authority over the study design; collection, management, analysis, and interpretation of data; writing of the report; or the decision to submit the report for publication.

## Introduction

### Background and rationale {6a}

Many health programmes experience large mismatches between those identified with a clinical need and those who attend services. A recent international systematic review of ‘no-show’ appointments across all medical specialities in primary and secondary care estimated that 23% of clinic appointments are not attended, with the highest rate observed in the African continent (43%) [1]. Complex supply and demand factors govern access to health services [2], and systematically marginalised populations are often the least likely to receive care [3, 4].

As mobile phone penetration has risen, there has been increasing interest in the use of phone-based reminder messages to reduce these missed appointments. Systematic reviews from 2011 [5], 2013 [6], 2016 [7], 2018 [8], and 2019 [9] have found that SMS and voice message reminders can improve clinic attendance by 50–100% depending on service, population and setting. In Linde and colleague’s systematic review of African RCTs, their pooled analysis found that SMS reminders doubled appointment attendance compared with no SMS (odds ratio 2.03; 95% confidence interval 1.40 to 2.95; *I*^2^ = 85%) [9]. Robotham and colleagues’ 2016 review found that two or more notifications increased attendance by as much as 19% over and above sending one notification, and voice notifications may offer slight improvements over text notifications for increasing attendance [7].

SMS and voice messages function as behavioural ‘brief interventions’, and a number of studies have used behavioural economics principles to guide the wording of these messages in order to optimise their impact [10, 11]. Senderey and colleagues used a set of established cognitive biases to develop the content of eight different clinic reminder messages [12] and Huf and colleagues used a similar approach in developing four different messages to boost clinic attendance in the UK [13]. Whilst both studies showed improvements in clinic attendance rates, neither provided the rationale for why these specific biases were selected. In Linde and colleagues’ 2019 systematic review of 31 African RCTs using phone-based messages [9], only one study reported using behavioural theories to develop the content of their reminder message: Erwin and colleagues successfully boosted cervical cancer screening rates among women in Tanzania [14], basing their SMS reminder content on the Health Belief Model [15]. They also reported using a motivational tone—found to be more effective than an informational tone [16]—and pre-tested the SMS content validity and cultural sensitivity of the message with programme staff and laypeople.

One area that currently experiences very high rates of missed appointments—with substantial societal and economic costs—is community-based vision screening. Approximately 1.1 billion people (over 10% of the global population) currently live with a form of easily correctable visual impairment [17, 18]. Two very cheap and simple interventions—spectacles and cataract operations—could eliminate over 90% of all visual impairment worldwide [17]. Provision of these services has risen exponentially in recent decades; however, effective coverage rates are disappointingly low and exhibit marked socioeconomic gradients at the international and intra-national levels [17]. Women and marginalised groups bear a disproportionate burden of visual impairment, and often face structural social barriers that prevent them from accessing care—as noted in the recent UN Resolution on Vision [19].

Recognising the human, social, and economic drag exerted by cataracts and uncorrected refractive error, many low- and middle-income country (LMIC) governments are ramping up their vision screening programmes. Donor funding is rising in tandem, partly driven by the advent of phone-based screening platforms like Peek Acuity [20, 21] that have made it possible to rapidly screen entire regions with very modest resource requirements.

Screening programmes based on the Peek digital platform are currently operating in seven LMICs. The Botswanan Ministry of Health (MoH) has committed to use Peek software to screen all school children in government schools over 3 years beginning in Summer 2022 [22]. The Peek platform records basic sociodemographic data, visual acuity, referral status, and attendance status for each child. Every time a child is referred a series of three SMS messages are sent to the mobile phone number registered by their parent/guardian (see Table 1). The current SMS message was not developed with reference to behavioural economics principles. According to internal data from Peek screening programmes in other countries and pilot data in Botswana, attendance rates are currently around 50%, i.e. only half of those identified as needing ophthalmic assessment present to services.

We aim to develop two behavioural economics-informed reminder messages; an SMS and a pre-recorded voice message to be used in the new Botswana MoH schools-based vision screening programme, and tested using an embedded, pragmatic, adaptive RCT design.

### Objectives {7}

Our objectives are to test a behavioural economics-informed SMS reminder message and a pre-recorded voice message that will be sent to the parents/guardians of school children who have been identified as having a visual impairment and referred to clinic. We hypothesise that these messages will be associated with a higher attendance rate than the current standard SMS reminder that is sent to all referred patients’ parents/guardians.

### Trial design {8}

This is an automated, adaptive, parallel, four-arm, embedded, pragmatic RCT. We will start by testing the two SMS reminder messages head-to-head with an initial 1:1 allocation ratio, and then introduce the voice messages after a period of six weeks. We will use a Bayesian adaptive trial algorithm to perform adaptive allocation as the trial progresses.

## Methods: participants, interventions and outcomes

### Study setting {9}

The Botswana National Comprehensive School Eye Health Program (‘Pono Yame’).

### Eligibility criteria {10}

Reminder messages will be sent to the registered mobile phone numbers of parents/guardians of children who test positive at screening and are referred on to clinic in the Pono Yame MoH/Peek Vision school screening programme in 2022. Provision of a mobile number is a pre-condition of entry into the screening programme, although parents/guardians are able to supply the number of a friend or relative so in practice this stipulation does not exclude any children.

Reminders will be sent in English and Setswana; spoken by >96% of the local population. The screening programme routinely collects data on preferred language, and reminders will be sent in the preferred tongue. Those who list any language other than English of Setswana will receive the reminder in both Setswana and English. The reminder will also be sent in both languages to those where data on language is not available for any reason. We will perform a secondary analysis that excludes these participants, but they will be included in the primary analysis.

### Who will take informed consent? {26a}

The interventions represent minor modifications to existing routine processes and present negligible risk to participants. Obtaining consent would introduce burdens to the participant that are greater than the intervention itself. As such, we will not seek informed consent. This approach has been approved by the LSHTM and University of Botswana ethics committees, and follows the precedent set by three previous RCTs testing SMS reminder messages [12, 13, 23].

### Additional consent provisions for collection and use of participant data and biological specimens {26b}

All parents/guardians are verbally informed that their children will partake in the Pono Yame vision screening programme. They are also asked to provide written opt-out consent for the use of their children’s sociodemographic data for research and sharing purposes. Care will not be compromised in any way for those participants whose parents do not provide consent.

### Interventions

#### Explanation for the choice of comparators {6b}

The standard SMS message presented in Table 1 is routinely sent to the registered mobile phone of parents/guardians of children referred on for refractive services in all Peek programmes. This is the control arm.

**Table 1:** Control and intervention reminder messages

**Control: Standard SMS reminder message** **Setswana** Go [name], one wa tlhatlhobiwa matlho mme ga fitlhelwa ona le bothata jwa matlho. Ka jalo, tla ko [location] ka di [date] go tlhatlhobiwa.O kopiwa go tla le karata ya gago ya botsogo. **English** Dear [name], you were examined and found to have an eye problem. Kindly report to [location] on [date] for assessment. Please come with your clinic card. **Intervention: New SMS reminder message** **Setswana** Go motsadi: Re lemogile ngwana wa gago [child’s name] fa ana le bothata jwa matlho. Se, se ka ama tiro ya gagwe ya sekolo. Tswee-tswee, tsisa [child’s name] ko sekolong ka [location and time] o tla tlhatlhobiwa matlho a sa duele Se, se direlwa ngwana mongwe le mongwe mo sekolong yoo nang le bothata jwa matlho Re ka leboga go le bona ka [day and time]. Kea leboga [Leina la ngaka] **English** Dear parent, we have found that your child [child’s name] has an eye problem. This may affect [his / her] schoolwork. Please bring [child’s name] to [location] at [time], [day, date]. We will be doing a free medical check-up for all the children with eye problems in the school. We look forward to seeing [child’s name] on [day and time]. Many thanks, Dr [name], Ministry of Health **Intervention: New voice reminder message** **Setswana** Dumelang: Ke bidiwa ngaka Dineo, go tswa ko lephateng la botsogo. Ngwana wa gago [leina la ngwna] o tlhatlhobilwe matlho mo bogaufing, mme a fitlhelwa a na le bothata jwa matlho. Fa a ka seka a alafiwa , go ka ama tiro ya gagwe ya sekolo. Setlhopha sa rona sa botsogo, se tlaa bo se le ko sekolong sa ga [leina la ngwana] ka [letsatsi le nako]. Tswee.tswee tsisa ngwana wa gago go tlhatlhobiwa go sena dituelo. Se, se direlwa ngwana mongwe le mongwe yoo nang le bothata jwa matlho. Kea leboga [Leina la ngaka] **English** Hello, my name is Dr [name] from the Ministry of Health. Your child [child’s name] recently had [his/her] eyes checked at school and was found to have an eye problem. If this is not corrected, it could affect their schoolwork. Our medical team will be at [location] on [date/time]. Please bring your child to get a free medical assessment. This is offered to all children with eye problems. We look forward to seeing you and your child on [date/time] Many thanks, Dr [name].	

### Intervention description {11a}

#### Process of developing the intervention SMS and voice reminder messages

We aimed to use an established framework to identify a theory-informed set of behaviour change principles to guide the development of our reminder messages. We elected to use Dolan and colleagues’ *MINDSPACE* framework [24], developed in conjunction with the Institute for Government. This framework brings together insights from behavioural economics research that can be used to develop brief healthcare interventions (Table 2). The framework has been endorsed by the Behavioural Science and Public Health Network [25], the London School of Economics Behavioral Economics Playbook for behaviour change [10] and the Health Foundation in their guidance on behavioural insights in health care [11].

**Table 2:** The ‘MINDSPACE’ framework and application for phone-based reminder messages

Principles	Application
Messenger	People are heavily influenced by the authority and credibility of the person sending the message, so the reminder messages should be signed-off by a trusted official/professional.
Incentives	People are more sensitive to losses than gains, so the reminders should frame non-attendance as a loss.
Norms	People want to fit in and are strongly influenced by the actions of others, so the reminders should signal that attendance is the norm.
Defaults	People tend to ‘go with the flow’ and use pre-set options, so attendance should be the default option in the reminders.
Salience	People are drawn to things that are novel and appear relevant to them, so the reminders should be personalised and stress the novelty of the opportunity.
Priming	Peoples’ decisions are commonly influenced by subconscious cues in their environment. We cannot influence this via phone-message.
Affect	Peoples’ decisions are often based on emotional associations rather than facts, so the reminders should seek to make emotive arguments for attendance.
Commitments	People seek to be consistent with public promises and reciprocate acts, so the reminders should aim to elicit a commitment to attend and stress the social expectation of attendance.
Ego	People act in ways that support the impression of a positive self-image, so the reminders should reinforce the message that attendance is consistent with recipients’ positive self-perceptions.

Whilst SMS and voice messages can include messenger, incentives, norms, defaults, salience, emotional appeals, commitments, and ego, they are less able to ‘prime’ recipients using subconscious cues. In addition to these principles, we also looked to the specific guidance on sending effective phone messages to reduce clinic non-attendance produced by Public Health England in 2020, based on their review of the international literature [26]. Their key messages are summarised below:

- Messages should be clear, brief and well-formatted, with essential information only.
- Use line breaks to make the message easier to read.
- Personalise the text messages to include the recipient’s name if local systems allow.
- Keep messages to 480 characters (3 standard text messages) in length.
- Include the date, time, and location of the appointment, as well as any special instructions, and contact phone number (if different to the number the text message is sent from).
- Write out the day of the week and the month in dates. For example, ‘Monday 23 March’.
- GP endorsement can encourage people to take screening more seriously.

**Fig. 1:**
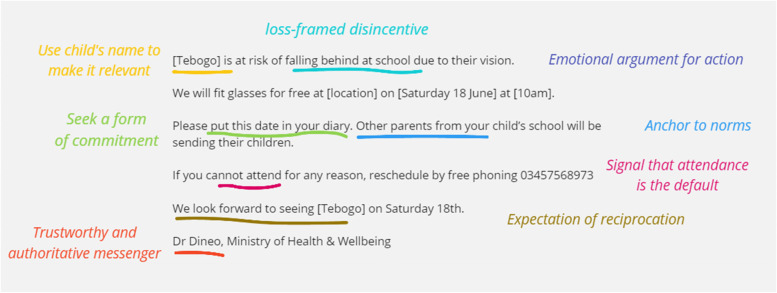
First draft SMS reminder. Note: ‘Tebogo’ and ‘Dr Dineo’ are not real names

One researcher (LA) drafted an initial SMS that included all of the eight relevant MINDSPACE behavioural economics elements and adhered to PHE guidance (Fig. 1). We convened a workshop to refine the SMS and develop a pre-recorded voice message with an African economist and representatives from the University of Botswana, and Peek Vision’s Botswana office. Further iterations were made following a robust refinement process (Additional file 1: Appendix 1) that included input from laypeople, and professional translation and back-translation. The final messages are presented in Table 1.

We will use four arms as outlined below. Each SMS will be sent two times; on the day of referral and on the day before the appointment.

Initially, we will only test arms 1 and 2 (the control and intervention SMS messages). We plan to introduce the voice message arms after 6 weeks. This is because we are interested in introducing new arms at later stages in the screening programme and want to observe how the allocation algorithm handles the introduction of new arms part-way through testing established interventions.

- Arm 1 (Control): Standard SMS reminder messages.
- Arm 2: New SMS reminder messages.
- Arm 3: Standard SMS reminder messages plus the pre-recorded voice reminder
- Arm 4: New SMS reminder messages plus the pre-recorded voice reminder

### Criteria for discontinuing or modifying allocated interventions {11b}

Due to the low-risk nature of the interventions, there will not be any formal option to discontinue or modify the reminder messages.

### Strategies to improve adherence to interventions {11c}

There are no relevant strategies to improve adherence. This is a pragmatic intention-to-treat study, and we will not collect data on whether messages were actually read or listened to by the intended recipients. A potential limitation of this study is that we cannot ensure that the message is actually delivered to- and read by the correct person. To an extent, this is true of all forms of phone-based reminder messages, as it is of paper reminder letters or notes sent by post, or home with children.

### Relevant concomitant care permitted or prohibited during the trial {11d}

No other reminder messages will be sent from the Peek platform during the trial.

### Provisions for post-trial care {30}

As this is a negligible risk trial, no provisions will be made for post-trial care.

### Outcomes {12}

All children who are screened and found to need further assessment and treatment (e.g. refractive services) will be given an appointment, approximately 1 week later, at a specified field ‘triage and treatment’ clinic or at a specialist ophthalmic hospital clinic. The primary outcome is attendance at this pre-specified appointment on the appointment date (yes/no).

The Peek software retains a record of every referred child. When children attend for these appointments, they are checked in using Peek software. This automatically updates their attendance status. Attendance data will be automatically reviewed by an algorithm every 24 h. The great advantage of the Peek-based screening programme is that is a closed data system with complete, unified data records for every person screened, their referral status and their attendance status. No additional data collection activities are required.

- *Primary outcome*: attendance at clinic on invited date. This is a binary outcome measure (yes/no). We will compare mean outcome rates between arms.
- *Secondary outcome*: days elapsed between appointment date and attendance. This is because children may miss their appointed day but attend at a later date. We will compare mean number of days elapsed between each arm.
- *Subgroup analyses*: attendance by age, sex, urban/rural residence, distance to clinic, ethnicity, guardianship, religion, language, household composition, migrant status, parental occupation, housing, assets and income.

### Participant timeline {13}

This automated adaptive trial will run continually for three working months (i.e. pausing during the school holidays when screening does not happen), recruiting participants until sufficient evidence has been gathered to reject the null (by triggering a stopping rule). Enrolment is planned to commence in quarter 3 2022. We intend to start with two SMS arms and add in voice messaging once the trial is underway. This is because we want to observe how the automated allocation system handles the introduction of new arms.

### Sample size {14}

Approximately 1000 children will be screened every day. Based on previous programmes, we expect approximately 160 of these children to be identified as requiring referral for further assessment and treatment. All of these children’s parents/guardians would receive the standard SMS reminders in a standard programme.

The adaptive allocation method that we are using does not use a pre-specified a sample size. Instead, the study will run until one of two criteria is met:

- There is a >95% probability that one arm is best.
- There is a >95% probability that the difference between the arms remaining in the study is <1%.

Depending on the effect of the interventions, one of the stopping criteria might be met after a few days; however, it could also take years before reaching a definitive conclusion. We will set a 3-month limit for this current study due to resource constraints.

### Recruitment {15}

Community sensitisation is being led by the Ministry of Health and Ministry of Education. This includes TV and radio coverage explaining the Pono Yame screening programme. Our field coordinator will visit each region and work with schools to ensure that they are set up to enrol as many children as possible. Every referred child’s data will be included in the primary analysis. Subgroup analyses will only be permitted for children whose parents have consented for their sociodemographic data to be used for research purposes. This is a separate consenting process led by Peek.

### Assignment of interventions: allocation

#### Sequence generation, concealment and implementation {16a, 16b, 16c}

Participants will initially be randomly allocated into two arms using computer-generated blocks of 12. As allocation and intervention delivery (sending SMS messages) is fully automated, there is no need for any of the human investigators to know participant allocation status. Once the first participants attend refractive services, the algorithm will begin adjusting the allocation ratio to favour the best-performing arms. There is no need for the investigators to see allocation status at this stage either. The data safety monitoring committee will be fully unmasked to allocation status and all outcome data and will have the power to stop the trial or suspend any arm.

### Assignment of interventions: blinding

#### Who will be blinded {17a}

Trial participants will not be blinded. Programme implementers will check in participants when they attend clinic using Peek Capture. The software will automatically record the date and the time elapsed since referral. The adaptive algorithm will analyse attendance rates between arms according to pre-defined rules. Screening programme staff and data analysts will be blinded to assignment status. A small team of unblinded human statisticians will monitor the algorithm’s performance. They will double-check the algorithm’s working every 24 h during the trial and will repeat the final analysis comparing each arm. They will have the power to stop the trial, but they will not influence allocation.

#### Procedure for unblinding if needed {17b}

There is no procedure for unblinding.

### Data collection and management

#### Plans for assessment and collection of outcomes {18a}

Referral status, attendance status and days elapsed since referral will be collected using the Peek Capture system on Android devices. Every time a participant is referred and every time they attend at clinic, they are checked in using an android device operating Peek Capture software. Additional data on sociodemographic characteristics will be collected when participants initially present to the screening programme.

#### Plans to promote participant retention and complete follow-up {18b}

As the intervention is an SMS sent automatically by the programme, there is no scope for deviation. Similarly ‘loss to follow-up’ is the reciprocal for our primary outcome (attendance on appointed day).

#### Data management {19}

Data will be collected by Peek’s implementing partners using Android devices through the Peek Capture application. Peek Capture enforces security controls that include strong device passcodes and native Android encryption. Data stored is time limited, the device syncs via an encrypted connection with a Peek-managed server, the data is then deleted to minimise the risk of data stored on the device.

Data will be stored on a Peek-managed server hosted in a Virtual Private Cloud (VPC) utilising the Amazon Web Services (AWS) Cloud. Each Peek-powered programme is hosted on its own dedicated server and a VPC that will reside in the UK/EU ensuring all of the data privacy safeguards as governed under the GDPR. All data collected is securely stored in AWS data centres which are state of the art, utilising innovative architectural and engineering approaches. Routine manual data cleaning will be conducted periodically by Peek administrators. Internal software guardrails will pick up simple errors.

Data collected can be monitored using Peek Admin; it tracks the Programme progress, provides insights and helps ensure no one is left behind. Data exported from Peek Admin will be pseudo-anonymised removing names and any other key identifiers, only the least amount of data will be shared, and where possible it will be fully anonymised and aggregated for research purposes.

At the analysis stage, data will be sent via a secure file transfer, using an encrypted zip file to LSHTM researchers to perform statistical testing. The zip file will be saved on the protected LSHTM server and only authorised named project staff will be given access. Passwords will be sent separately. Further details can be found in the Data Management Plan (Additional file 1: Appendix 2).

#### Confidentiality {27}

Peek routinely collects sociodemographic information from each child who is referred on for refractive services including age, sex, location, ethnicity, religion, parents' occupation, parents' education, housing characteristics and asset ownership. This information will be held on a Peek-managed server hosted in a Virtual Private Cloud (VPC) utilising the Amazon Web Services (AWS) Cloud. Peek also seeks consent to use this data for research purposes.

Sociodemographic data on participants who have provided consent will be shared with the statistical analysis team at LSHTM for subgroup analysis. All team members who will access these data will have undertaken information security training. We will use encrypted data transfer and avoid cloud services outside the EU. The aggregated Peek data that is shared with LSHTM project staff will not contain any names; however, the data being shared may still permit the identification of individuals depending on the domains being shared and may therefore constitute pseudo-anonymised data. All data arising from this project will be stored securely for 10 years. Further information is provided in the data management plan (Additional file 1: Appendix 2).

#### Plans for collection, laboratory evaluation and storage of biological specimens for genetic or molecular analysis in this trial/future use {33}

Not applicable. We will not be using biological specimens.

### Statistical methods

#### Statistical methods for primary and secondary outcomes {20a}

**Fig. 2:**
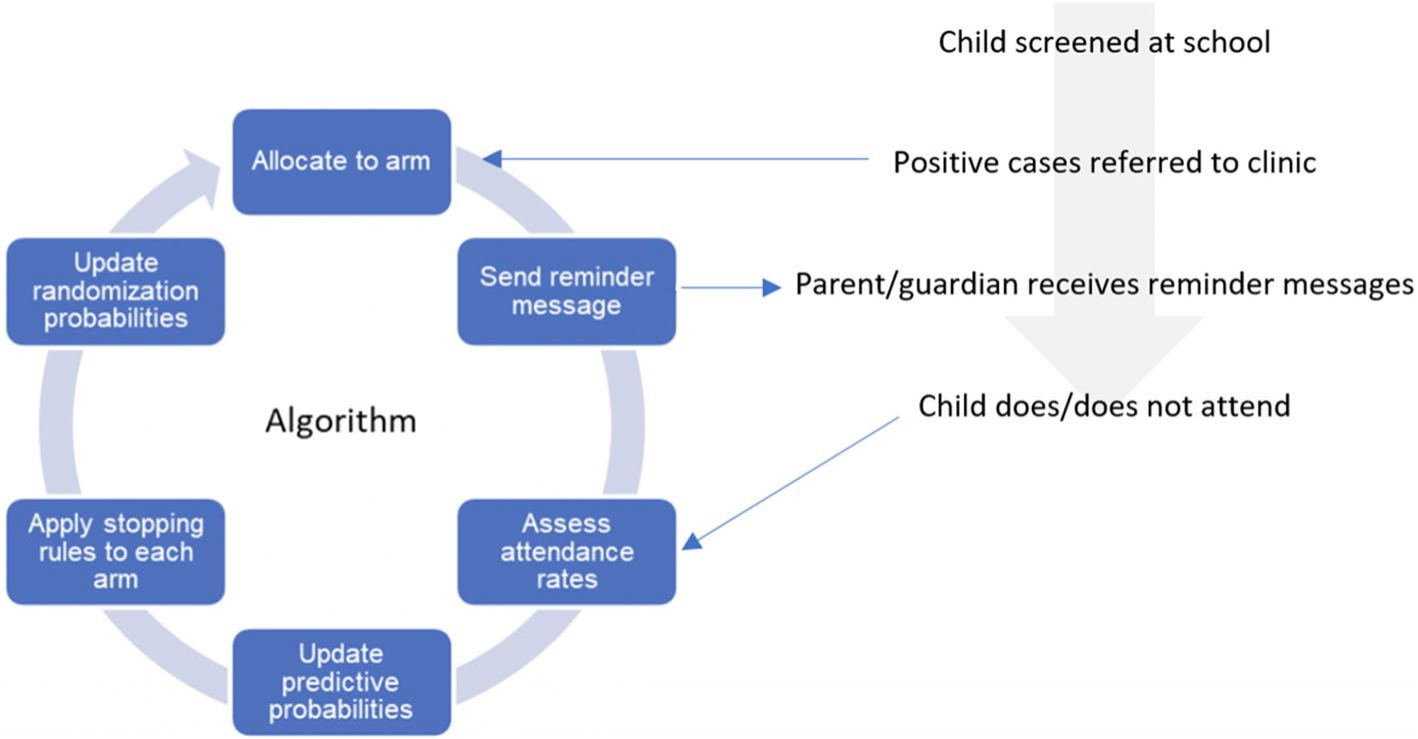
Interaction between patient flow and the adaptive trial algorithm

This study will use Thompson sampling a Bayesian approach to identify the best arm. This is a Bayesian algorithm widely used to learn about arms and optimise decision making [27] Every 24 h, the probability of each arm being the best arm overall will be estimated, using Monte-Carlo simulations to get the posterior probability estimates. As there is no evidence available on how the messages would perform relative to another, a regularising prior of Beta(100,000) (i.e. centred at *p*=0.5 with a 90% credible interval of 0.44–0.56) will be used to avoid overfitting extreme data in the early phase of the trial. It is expected that about 1,000 children will enrol every day, and the observed data will begin to dominate the prior within the first couple of days. Each arm will have a probability of being best between 0 and 100%, and the sum of all two probabilities will equal 100%. These probabilities will be compared to the stopping rules as to whether the trial should stop or continue into the next day. If the trial is to continue, the proportion allocated to each arm for the next day will be updated to be proportional to the estimated probabilities. We will conduct all analyses using the runif and rbeta functions in R (R Foundation for Statistical Computing, Vienna, Austria). Figure 2 illustrates participant flow and operation of the algorithm.

#### Interim analyses {21b}

This is an automated adaptive trial. Our algorithm will review the attendance data every 24 h and perform statistical testing. Two stopping rules will be applied during these daily interim analyses:

1. There is a >95% probability that one arm is best.
2. There is a >95% probability that the difference between the arms remaining in the study is <1%.

**Fig. 3:**
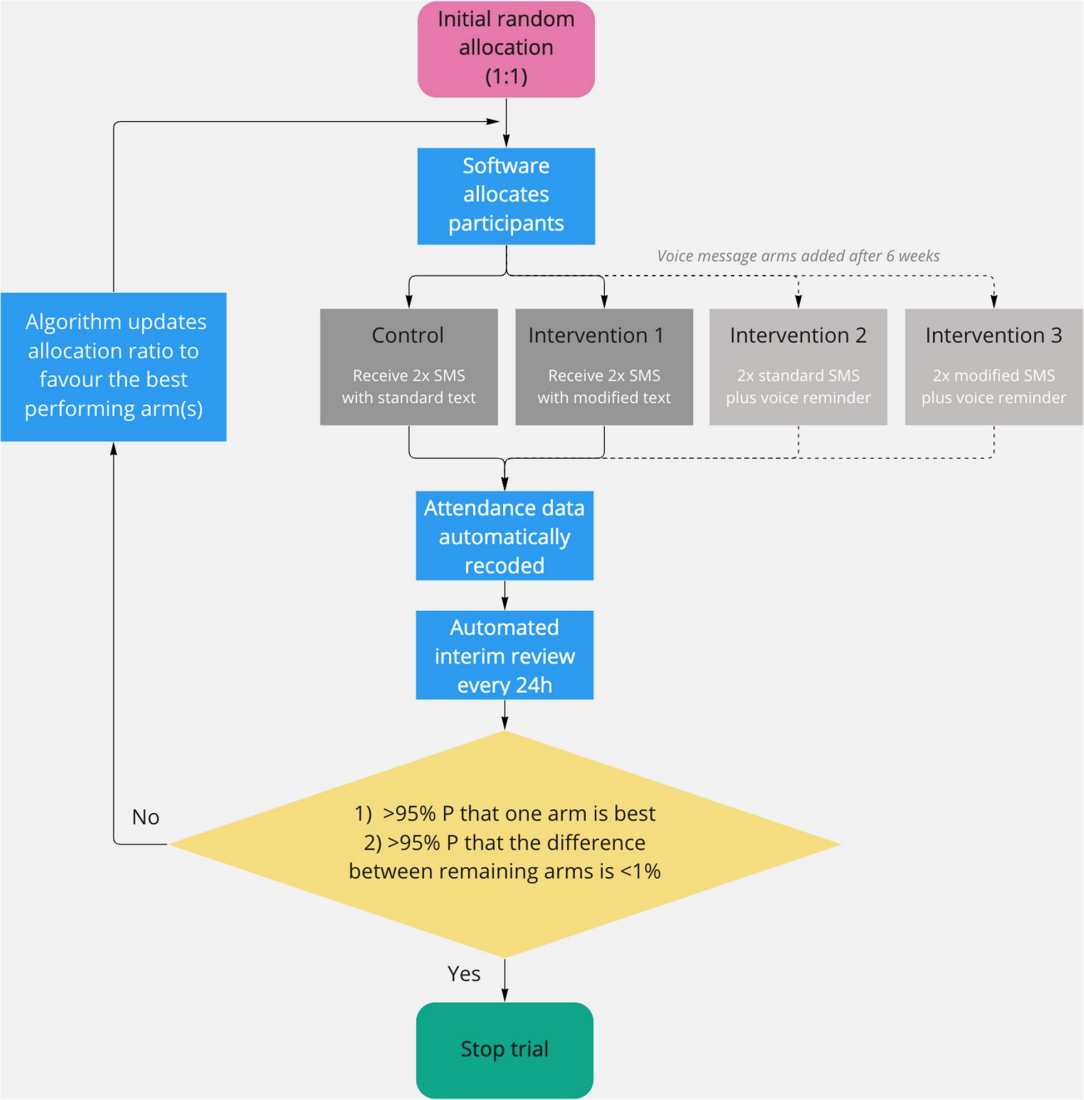
Allocation flow diagram

If neither of these rules have been satisfied, then the trial (i.e. enrolment) will continue until three months of active screening have elapsed. The Bayesian algorithm will adjust the allocation ratio based on the performance of each arm with respect to the updated posterior probability that each is associated with attendance (Fig. 3).

#### Methods for additional analyses (e.g. subgroup analyses) {20b}

Internal data from a pilot site suggests that around 10% of children who attend the ‘triage and treatment’ clinic will subsequently be identified as having an eye need that requires further specialist ophthalmological assessment in a hospital clinic. These children will be referred from the ‘triage and treatment’ clinic to the local hospital. This subgroup will also receive either the intervention or control reminder messages. Again, the outcome will be attendance on appointed date.

Once the trial is complete, we will perform retrospective subgroup analyses to explore whether attendance within each group was associated with sociodemographic variables. We use multivariable logistic regression to assess whether each sociodemographic variable is associated with attendance. We note that this is an exploratory analysis, providing hypotheses that can be tested in subsequent studies.

We will perform a secondary analysis that excludes participants whose preferred language is neither Setswana nor English.

#### Methods in analysis to handle protocol non-adherence and any statistical methods to handle missing data {20c}

The primary analysis only requires trial arm and the outcome (attendance) to be recorded. The trial arm should be recorded automatically as part of the Peek coding, and if it was missing it would be due to a bug in the coding. If this occurred, there is no statistical method that could be used to recover that data so any records with trial arm missing would not be included in the updating of the probability that an arm is best. We will check the code every 24 h to ensure that it is running as expected and correct any errors that we find immediately. The outcome cannot be missing, as a participant is set as ‘not attended’ until the point where they are updated as having ‘attended’.

#### Plans to give access to the full protocol, participant-level data and statistical code {31c}

The full protocol is available from the corresponding author. Statistical code will be made freely available online using GitHub. In line with the UK concordat on open research data (2016), anonymised participant-level data from this trial will be made available to bona fide research groups (evidenced via curriculum vitae and the involvement of a qualified statistician), and in line with the trial’s publicly available data sharing policy, following review and approval from the trial’s data monitoring committee. No reasonable request will be turned down, and the appropriate data will be made available within 1-month of receiving the request. There may be multiple levels of permission required in-country before data can be shared, including national ministry of health approval and local implementation partner approval.

### Patient and public involvement

Laypeople were involved in checking the wording of the intervention messages and suggesting refinements that better conveyed their underlying meaning.

### Oversight and monitoring

#### Composition of the coordinating centre and trial steering committee {5d}

Trial coordinating centre:

- Dr Luke Allen, Co-Principle Investigator and trial manager, LSHTM
- Hannah Chroston, lead administrator, LSHTM
- Bakgaki Ratshaa, trial coordinator, University of Botswana

Trial management group

- Prof Andrew Bastawrous, chief investigator
- Prof Oathokwa Nkomazana, co-PI
- Dr Luke Allen, co-PI
- Prof Matthew Burton, methods advisor
- Dr David Macleod, lead statistician
- Dr Nigel Bolster, Peek integration
- Min Kim, statistician
- Dr Ari Ho-Foster

Dr Michael Gichangi, methods advisor

Data management team

- Dr Luke Allen, co-PI
- Dr David Macleod, lead statistician
- Dr Nigel Bolster, Peek integration
- Min Kim, statistician

#### Composition of the data monitoring committee, its role and reporting structure {21a}

An independent Data and Safety Monitoring Board (DSMB) will be appointed by the trial steering committee. The DSMB will have three members, all independent of the running of the trial with relevant clinical and epidemiological experience.

The DSMB will confirm their specific meeting arrangements. It is proposed that the DSMB would meet prior to the beginning of the trial (Q2 2022), one third of the way through, and at the end, to assess the safety of the trial procedures. The DSMB will agree the way it will monitor the data, what it requires from the investigators in this respect and will communicate this to the PIs. All data can be interrogated remotely in real time.

The DSMB may visit the study coordination centre to assess data management, record keeping and other important activities. The DSMB will determine the manner in which it will monitor the data, what it requires from the investigators in this respect and will communicate this to the PIs.

### Adverse event reporting and harms {22}

#### Definitions

**Table:** 

**Term**	**Definition**
Adverse event (AE)	Any untoward medical occurrence in a patient or study participant
Serious adverse event (SAE)	A serious event is any untoward medical occurrence that: Results in death Is life-threatening Requires inpatient hospitalisation or prolongation of existing hospitalisation Results in persistent or significant disability/incapacity Consists of a congenital anomaly or birth defect Other ‘important medical events’ may also be considered serious if they jeopardise the participant or require an intervention to prevent one of the above consequences.

#### Reporting procedures

All adverse events will be reported. Depending on the nature of the event the reporting procedures below will be followed. Any questions concerning adverse event reporting will be directed to the study coordination centre in the first instance. The flow chart below has been provided to aid the reporting of AEs.

#### Responsible personnel

##### Chief Investigator (CI)

- The CI has overall responsibility for the conduct of the study and the ongoing safety and evaluation of any IMPs being used in the trial.
- Promptly notifying all investigators, Institutional Review Board (IRB) or Independent Ethics Committee (IEC) and Competent Authorities (CAs) of each concerned member state of any findings that may affect the health of the trial participants.
- Keeping detailed written reports of all AEs/ARs identified in the protocol as critical to the evaluation of safety within the agreed timeframes specified in the protocol.
- Accurate production and submission of the Development Safety Update Reports and progress reports to CAs and IRB/IECs.
- Collate all AR/AEs/SAEs/SARs and report to the Sponsor annually.
- Ensure that the PIs report all SAEs/SUSARs immediately to the Sponsor and to the CAs, IRB/IECs and any other relevant parties within agreed timelines
- Supplying the Sponsor and IRB/IEC with any supplementary information they request.

##### Principal Investigators (PI)

- The PIs have responsibility for the research performed at the local site, handling and management of investigational medical products, and informing the CI, Sponsor, Ethics, regulatory bodies and the trial coordinating team, of all adverse events that occur at their site
- Safety responsibilities:
- Ensure trial participant safety and the swift and adequate management of trial participants with any type of AE/AR as per the management protocol described below.
- Reporting all SAEs/SUSARs immediately to the Sponsor and to the CAs, IRB/IECs and any other relevant parties within agreed timelines (i.e. LSHTM, EFMHACA, ORHB, FMOST).
- Assessing each event for causality, severity and expectedness. (Note: a medical decision which must be made by the investigator directly involved with the care of the patient/participant experiencing the AE)
- Ensure adequate archiving of AE records and reports in the local trial office along with the trial master files.
- Collate all AR/AEs/SAEs/SARs biannually and present to the CI.
- Guide and supervise the field research team on accurate recording, reporting of all adverse events.

##### Field Research Team Members (Coordinators, Nurses, Examiners, Recorders)

- All field research team members are responsible for identifying, recording and reporting any AE or AR to the PIs regardless of severity or causality.
- Assessing each event for causality, severity and expectedness. (Note: a medical decision which must be made by the investigator directly involved with the care of the patient/participant experiencing the AE).
- Ensure that the participant has received the necessary management. This includes advice/reassuring, referral, offering transport, paying for management, making follow-up visits
- Report to the PIs/Project manager AEs/ARs based on the specified timeline and file all AE/AR recorded forms in the trial master file.

##### Non-serious AEs

All non-serious AEs will be reported to the study coordination centre and recorded in a dedicated AE log within 72 h. The entry must state the patient ID, date and time of AE, nature and relation to the intervention, if any. The AE should also be reported to the data and safety monitoring committee within 72 h. AE logs will be stored on a secure, password-protected file on a LSHTM computer.

##### Serious AEs

Serious adverse events (SAEs) will be reported to the PI and study coordination centre within 24 h of the local site being made aware of the event. The PI will report the event to the data safety monitoring committee within 48 h and include it in the study safety report.

An SAE form will be completed and submitted to the PA and study coordination centre with details of the nature of event, date of onset, severity, corrective therapies given, outcome and causality. All SAEs whether expected, suspected or unexpected will be reported to regulatory bodies and the trial DSMB within 48 h of occurrence. The responsible investigator will assign the causality of the event. All investigators will be informed of all SAEs occurring throughout the study. If awaiting further details, a follow-up SAE report should be submitted promptly upon receipt of any outstanding information.

Any events relating to a pre-existing condition or any planned hospitalisations for elective treatment of a pre-existing condition will not need to be reported as SAEs.
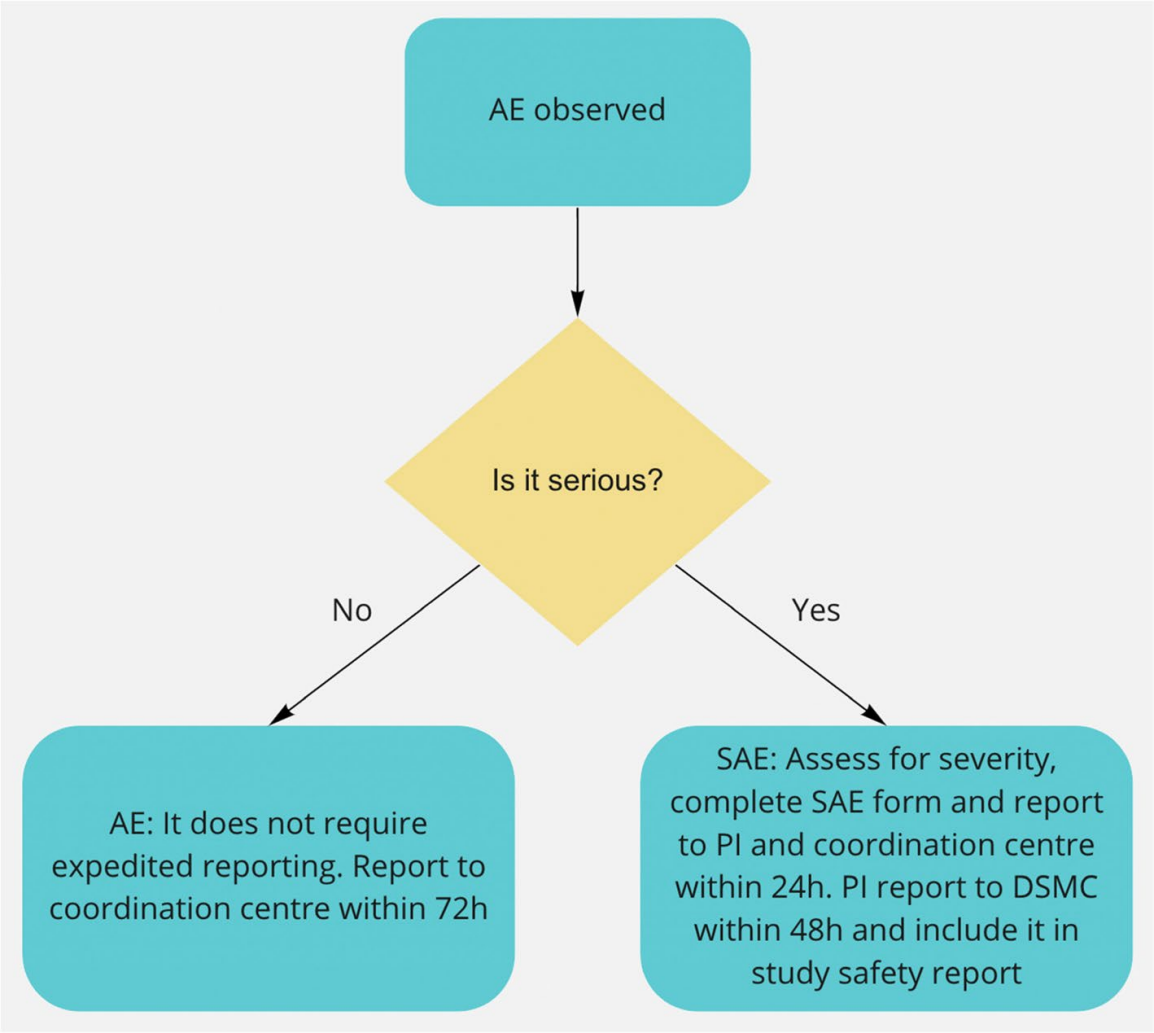


Contact details for reporting SAEs

Please send SAE forms to: luke.allen@lshtm.ac.uk or nkomazanao@UB.AC.BW using the title ‘SAE’

Tel: +44 (0) 20 7958 8316 (Mon to Fri 09.00–17.00)

Tel: +267 355 0000

#### Frequency and plans for auditing trial conduct {23}

The study may be subject audit by the London School of Hygiene & Tropical Medicine under their remit as sponsor, the Study Coordination Centre and other regulatory bodies to ensure adherence to Good Clinical Practice.

#### Plans for communicating important protocol amendments to relevant parties (e.g. trial participants, ethical committees) {25}

Any important protocol modifications will be reported to the co-investigators, research committees, the trial registry and—where appropriate—journals and regulators via email.

#### Dissemination plans {31a}

Scientific results will be published in Open Access in peer-reviewed journals and presented at relevant international conferences. All publications and presentations relating to the study will be authorised by the Trial Management Group. The first publication of the trial results will be in the name of the Trial Management Group members. Members of the Data and Safety Monitoring Board will be listed and contributors will be cited by name if published in a journal where this does not conflict with the journal’s policy. Authorship of any parallel studies initiated outside of the Trial Management Group will be according to the individuals involved in the project but must acknowledge the contribution of the Trial Management Group and the Trial Coordinating Centre.

## Discussion

This study is embedded in the national Pono Yame school-based vision screening programme. As such, any delays to the launch of the programme will delay the start of the trial. As far as we are aware, only one other study has used behavioural economics to inform the development of reminder messages to be deployed in an African healthcare setting. Our study will use an adaptive trial design, embedded in a national screening programme. Our approach can be used to trial other forms of reminder message in the future, including tweaks to the messages that are sent and varying message content according to the demographic characteristics of the recipient.

### Trial status

This is protocol version 1.2 (14 June 2022). Recruitment has not yet commenced but is planned for Q3 2022.

## Supplementary Information

**Additional file 1.**

## Data Availability

Any participants’ identifiable personal data collected following informed consent by the Trial Coordinating Centre will be stored in a secure Peek Vision server (See Data Management Plan for more details on the purpose and type of data collected, and the security of the Peek Vision server). Confidentiality protected in accordance with the Data Protection Act 2018 and the General Data Protection Act. Patient-level data will be pseudo-anonymised removing names and any other key identifiers before it is shared. Only the least amount of data will be shared, and where possible it will be fully anonymised and aggregated. All published findings will be at anonymous aggregate subpopulation level. In line with the UK concordat on open research data (2016), anonymised data from this trial will be made available to bona fide research groups (evidenced via CVs and the involvement of a qualified statistician), and in line with the trial’s publicly available Data Management Plan (Additional file 1: Appendix 2), following review and approval from the trial’s data monitoring committee. No reasonable request will be turned down, and the appropriate data will be made available within 1-month of receiving the request. Peek Vision has a signed data sharing agreement with the Ministry of Botswana that governs the use of patient data collected and used in Peek Vision screening programmes. There may be multiple levels of permission required in-country before data can be shared, including national ministry of health approval and local implementation partner approval.

## Abbreviations

DSMC: Data and Safety Monitoring Committee
LSHTM: London School of Hygiene and Tropical Medicine
RCT: Randomised controlled trial
WHO: World Health Organization
