# Supplementary material for: Protocol for an automated, pragmatic, embedded, adaptive randomised controlled trial: behavioural economics-informed mobile phone-based reminder messages to improve clinic attendance in a Botswanan school-based vision screening programme
Source: Trials. 2022 Aug 15;23:656. doi: 10.1186/s13063-022-06519-y (PMC9377141; doi:10.1186/s13063-022-06519-y)
Supplement: Supplementary file 1 — Additional file 1. [file 13063_2022_6519_MOESM1_ESM.docx]

**Appendix 1: Intervention refinement process**

***Refinement process***

The working group felt that the wording needed to be simplified so that parents/guardians with low levels of education would understand the messaging. The first sentence and the concept of ‘falling behind’ were felt to be too complex. We broke this sentence into two parts and simplified the language. The Peek team noted that refractive error is not the only problem that is managed, so we changed the wording around glasses to ‘eye problems’ to be more inclusive. The Botswanans in the group felt that we should drop the request to put the date in a diary as this may not be standard practice in many settings. We discussed whether the wording should request that participants make a commitment by responding to the SMS with the word ‘yes’, however this would incur costs on their behalf. Our economist – who also reviews ethics applications – felt that mentioning the behaviour of other parents may be felt to represent coercion rather than persuasion. We agreed on a compromise by stating that the screening service will be provided ‘for all children’. The group felt that ‘toll free’ was better wording than ‘free phoning’ and agreed that the default – and simplest option – should be to bring the child to the appointment. The group agreed to retain the final sentence in order to exert emotional reciprocity. It was agreed that a doctor would be the most appropriate authoritative messenger – rather than a professor or any other profession - and that the MoH represents the most authoritative and respected affiliate institution. We agreed to use the same wording for the voice message (Box 1).

**Box 1: Second draft following the workshop**

Hello, my name is Dr Dineo and I am calling from the Ministry of Health.

Your child recently had their eyes checked at school by the Pono Yame screening programme.

Unfortunately, your child was found to have an eye problem.

If this is not corrected, it could affect their schoolwork.

Our medical team will be at your child’s school on [date/time].

Please bring your child to get a free medical assessment.

If you cannot come on that day, please call us to reschedule;

We will text you a toll-free number that you can call.

If you can attend, there is nothing you need to do. Just bring your child on the day.

We are offering this free assessment to all children with eye problems.

We look forward to seeing you and your child on [date/time]

Many thanks, Dr Dineo.

The message was then sent round for review from the wider research team. Wording revisions were suggested and agreed by consensus. Both messages were then translated into Setswana and back-translated into English, and checked by team members fluent in English and Setswana.

The final draft text was then tested with three Batswana laypeople (two teachers and a parent of a child with spectacles) using a qualitative ‘think aloud’ approach.[1]] Each person was asked to read the messages and verbalise their thoughts. They were then asked to provide specific feedback to clarify and improve the wording. They recommended merging two sentences into one:

**Original**: *“We will do a free medical check -up for [name of a child]. We are doing this for all children with eye problems in the school.”*

**Recommended wording**: *“We will be doing a free medical check-up for all the children with eye problems in the school.”*

This feedback was incorporated into the final revision of the SMS and voice reminder messages by the original workgroup. The final intervention messages were translated and back-translated. The Setswana version was checked by Setswana speakers and the English back-translation was checked by two bilingual people. The final messages were approved by the research team and are presented in Box 2, alongside the standard (control) SMS. We note that PHE recommend aiming to fit the entire message into three 160 character SMS messages, however our message spills into four.

**Box 2: Control and intervention reminder messages**

**Control: Standard SMS reminder message**

**Setswana**

[name], [child’s name] o ne a tlhatlhobiwa kwa sekolong, a fitlhelwa a na le mathata a matlho. Tswe tswe mo tlise ko [location] go bona ba bongaka jwa matlho.

**English**

Dear [name], [child’s name] was examined at school and found to have an eye problem. Kindly report to [location] to see an eye specialist.

**Intervention: New SMS reminder message**

**Setswana**

Go motsadi:

Re lemogile ngwana wa gago [leina la ngwana] fa ana le bothata jwa matlho. Se, se ka ama tiro ya gagwe ya sekolo. Tswee-tswee, tsisa {leina la ngwana} ko sekolong ka ( letsatsi le nako)

( Leina la ngwana) o tla tlhatlhobiwa matlho a sa duele

Se, se direlwa ngwana mongwe le mongwe mo sekolong yoo nang le bothata jwa matlho

Fa, o ka se kgone go tla, o ka rulaganya letsatsi le sele ka go leletsa mogala wa mahala wa (xxxxxxxxx).

Re ka leboga go le bona ka [letsatsi le nako]

**English**

Dear parent, we have found that your child [child’s name] has an eye problem. This may affect [his / her] schoolwork.

Please bring [child’s name] to [location] at [time], [day, date]. We will be doing a free medical check-up for all the children with eye problems in the school.

If your child cannot come for any reason, please call us for free on number [xxxxxxxxxxxx].  We look forward to seeing [child’s name] on [day and time].

Many thanks, Dr [name], Ministry of Health

**Intervention: New voice reminder message**

**Setswana**

Dumelang: Ke bidiwa ngaka Dineo, go tswa ko lephateng la botsogo. Ngwana wa gago [leina la ngwna] o tlhatlhobilwe matlho mo bogaufing, mme a fitlhelwa a na le bothata jwa matlho. Fa a ka seka a alafiwa , go ka ama tiro ya gagwe ya sekolo. Setlhopha sa rona sa botsogo, se tlaa bo se le ko sekolong sa ga [leina la ngwana] ka [letsatsi le nako].

Tswee.tswee tsisa ngwana wa gago go tlhatlhobiwa go sena dituelo. Se, se direlwa ngwana mongwe le mongwe yoo nang le bothata jwa matlho. Fa o ka seka wa kgona , tswee-tswee ikgolaganye le rona ko mogaleng wa [TBD xxxxxx] go re neela letsatsi lesele . Kea leboga [Leina la ngaka]

**English**

“Hello, my name is Dr [name] from the Ministry of Health.

Your child [child’s name] recently had [his/her] eyes checked at school and was found to have an eye problem. If this is not corrected, it could affect their schoolwork. Our medical team will be at [location] on [date/time]. Please bring your child to get a free medical assessment. This is offered to all children with eye problems.

If you cannot come on that day, please call us to reschedule on (XXXXXX). We will send you this number in a text message.

We look forward to seeing you and your child on [date/time]

Many thanks, Dr [name].

Reference

1. Charters E. The Use of Think-aloud Methods in Qualitative Research An Introduction to Think-aloud Methods. Brock Education Journal [Internet]. 2003 Jul 1 [cited 2022 Jan 28];12(2). Available from: https://journals.library.brocku.ca/brocked/index.php/home/article/view/38

**Appendix 2: Data Management Plan**

Digital resources

Relevant details to mention: topics covered, type (e.g. survey), source (collected by self or others), format (e.g. STATA) and amount (e.g. 10 interviews). Draw attention to human or other data that require additional protection.

**Data and Data Collection Process**

The data will be collected in Peek powered Eye Health School and Community Programmes using Peek’s Capture application. During the Programmes initial screening process only basic and non personal identifying data is collected.

**Data Fields Collected During the Initial Screening Process:**

- Age
- Gender
- Awareness (optional)
- Spectacle status
- Diabetes status (optional)
- Visual Acuity or pass/fail threshold
- Eye Condition

A representative sample of those screened will also be asked to provide sociodemographic data to enable us to monitor the equity performance of our programmes e.g. are certain ethnic groups more likely to be screened? The additional sociodemographic indicators are:

- Ethnicity
- Marital Status
- Religion
- Migrant/refugee status
- Occupation
- Education
- Food adequacy
- Housing (floor material)
- Asset ownership

Based on the visual acuity threshold set prior to screening the Peek Capture automatically informs the data collector whether the attendee may potentially need onward treatment. For those screened negative no further data is collected. Only for those screened positive is further information collected. This ensures data collection is kept to an absolute minimum maintaining privacy and ensuring compliance with data protection regulations.

For those screened positive additional information is collected, but the data is always minimised to ensure only the required data is collected at each stage of the service, as described below.

**Data Fields Collected for Those Screened Positive at Triage:**

- Name
- Telephone number
- email address (optional)
- Visual Acuity

**Further Data Fields Collected for Onward Treatment:**

- Eye Condition
- Prescription
- Diagnosis

We will also collect the sociodemographic details of all those referred on for further treatment:

- Ethnicity
- Marital Status
- Location (urban/rural)
- Religion
- Migrant/refugee status
- Occupation
- Education
- Food adequacy
- Housing (floor material)
- Asset ownership

**Data Collection Tools:**

Android Mobile Devices - Data will be collected by Peek’s implementing partners using Android devices through the Peek Capture application. Peek Capture enforces security controls that include strong device passcodes and native Android encryption. Data stored is time limited, the device syncs via an encrypted connection with a Peek managed server, the data is then deleted to minimise the risk of data stored on the device.

**Data Storage:**

The data is stored on a Peek managed server hosted in a Virtual Private Cloud (VPC) utilising the Amazon Web Services (AWS) Cloud. Each Peek powered programme is hosted on it’s own dedicated server and a VPC that will reside in the UK/EU ensuring all of the data privacy safeguards as governed under the GDPR. All data collected is securely stored in AWS data centers which are state of the art, utilising innovative architectural and engineering approaches.  More information, including a virtual tour, can be found by visiting the link below.

<https://aws.amazon.com/compliance/data-center/>

Hardware and software

**Software**:

- Peek Capture - is an application that runs on Android devices that supports eye health screening and referral pathways to treatment
- Peek Admin - is a web based data platform application that is used to view the data collected by Peek Capture, it tracks the Programme progress, provides insights and helps ensure no one is left behind.
- STATA and R, and Excel will be used to analyse the data exported from Peek Admin

**Hardware**:

- Peek servers are hosted on Amazon Elastic Compute cloud-based virtual machines running Amazon Linux.
- Android devices,  locally managed by Peek’s implementing partners.

Data-related activities

| **Task** | **Description** |
| --- | --- |
| Start gathering SES data | In month 1 we will start gathering sociodemographic data from:   - a representative sample of all those presenting to be screened - all those identified with an eye care needs and referred on for treatment     These data will be transferred from Android devices in the field to Peek Admin, hosted on AWS.    Note that Peek programmes run continuously and we intend to gather data from participants in every programme so that we can promote equitable service delivery. |
| Clean SES data | Routine manual data cleaning will be conducted periodically by Peek administrators. Internal software guardrails will pick up simple errors |
| Analyse SES data | Every month we will perform simple descriptive statistical analysis of presentation rates and treatment attendance rates by SES category.    The output of this analysis will be anonymised and presented as mean attendance rates for each SES subgroup e.g. males x%, females z%. |

Quality checks

Outline any quality checks to be performed before, during and after the above activities, e.g. to ensure data are captured correctly, remain accurate and complete, or ensure you avoid recognised problems. The UK Data Services offers guidance at <http://ukdataservice.ac.uk/manage-data/format/quality.aspx>.

- Errors are flagged at the point of data entry by software that only accepts pre-specified responses e.g. phone numbers must be comprised of a set string length of digits.
- The software has built-in logic steps
- We will institute training and supervision for all data collectors
- Application logging, audit trails and alerting direct administrators to given issues post-collection e.g. when SMS messages fail to be delivered
- Post-collection human data checking using the Peek Admin programme e.g. for ID disambiguation

Ethical & legal issues

Local permissions for Peek powered eye health programmes are already in place. This is in the form of data processing agreements with Peek and the local MoH and/or local implementing partner. This provides a legal agreement between the parties that the data can be collected and processed. The proposed research will be authorised by the same parties to ensure full transparency and the data collection and processing will be managed under the same data processing agreement.

Informed consent is not required to run the phone-based reminder trial. LSHTM ethics board have approved this approach. We will not commence until the protocol has been approved by the University of Botswana ethics board, and approval from the government (HRU) board.

**Consent processes used for other elements of the screening programme and embedded research**

The Peek programme routinely collects sociodemographic data on all participants. This is independent of the current RCT. For this SES data collection we obtain written informed consent to collect, analyse, and publish anonymised aggregate participant data in peer-reviewed journals and online open-access data repositories. Individuals will not be identifiable. In line with UK guidance on risk-adapted approaches to obtaining informed consent, participants provide consent by ticking a box underneath the following statement:

*“I understand that my anonymous data may be shared with other researchers or online. I understand that I will not be identifiable from this information. I understand that my decision will not affect the care that I receive, and I am free to change my mind anytime I like.”*

Consent is obtained when participants initially present for screening. For screening programmes that include children (<18 years), we will seek consent from their parents/legal guardians using the following statement, sent home on a paper form along with the generic participant information leaflets:

*“I understand that my child’s anonymous data may be shared with other researchers or online. I understand that my child will not be identifiable from this information. I understand that my decision will not affect the care that my child receives, and I am free to change my mind anytime I like.”*

Approval will be sought from research ethics committees at LSHTM and each of the countries where screening takes place.

Documentation

- Standard operating procedures and an overall study protocol will be developed in line with LSHTM research guidance to cover all aspects of the research project.
- Standardised online training modules have been delivered for programme implementing partners tasked with data collection in the field.
- Training will be delivered to all project staff to ensure that they understand the requirements and are able to follow the SOPs.
- We have a data compendium which describes the custom sociodemographic variables that we will collect in each country, available [here](https://docs.google.com/document/d/1-dpJq6lz0OHOGZpEqLv2HayCO-ZgvcZ6/edit?usp=sharing&ouid=102548958613678845262&rtpof=true&sd=true).

**STORAGE AND SECURITY**

**Pre research data collection and storage in Peek powered eye health programmes**

The data will be collected in Peek powered Eye Health School and Community Programmes using Peek’s Capture application.  Data will be collected by Peek’s implementing partners using Android devices through the Peek Capture application. Peek Capture enforces security controls that include strong device passcodes and native Android encryption. Data stored is time limited, the device syncs via an encrypted connection with a Peek managed server, the data is then deleted to minimise the risk of data stored on the device.

The data is stored on a Peek managed server hosted in a Virtual Private Cloud (VPC) utilising the Amazon Web Services (AWS) Cloud. Throughout the eye health programme life cycle only approved implementation partners and Peek team members have access to programme data. Access is strictly controlled through the Peek Admin web based data platform application. This is used to view the data collected by Peek Capture, it tracks the Programme progress, provides insights and helps ensure no one is left behind.  **Peek Capture security:**

- Peek Capture is installed on implementing partners managed Android devices
- Peek Capture enforces security controls that include strong device passcodes and native Android encryption.
- Data stored is time limited, the device syncs via an encrypted connection with a Peek managed server, the data is then deleted to minimise the risk of data stored on the device.

**Peek Admin security:**

- Strong passwords, minimum of 12 characters, password strength meter where only ‘strong’ is accepted, blacklist passwords are enforced to ensure easily guessed and passwords found in data breaches cannot be used.
- 2-Factor Authentication to protect user account security.
- User access permissions are controlled through account privileges, this controls scope of programme so access is restricted and limited to only what a user requires for their work, admin privileges are restricted to only those that require the access, account management and patient level reporting.
- Accounts disable automatically after 60 days of inactivity.
- User access reviews available for implementing partners to ensure leavers and inactive accounts are removed.

**Peek Platform Data Security Assurance:**

Peek is an International Standardisation Organisation (ISO) 27001 certified organisation. ISO 27001 certification requires an annual audit by an accredited external auditing body who verify compliance with the industry best practice information security controls.

Peek servers hosted in a Virtual Private Cloud (VPC) utilising the Amazon Web Services (AWS) Cloud. Each Peek powered programme is hosted on it’s own dedicated server and a VPC that will reside in the UK/EU ensuring all of the data privacy safeguards as governed under the GDPR. All data collected is securely stored in AWS data centers which are state of the art, utilising innovative architectural and engineering approaches.

More information, including a virtual tour, can be found by visiting the link below:

<https://aws.amazon.com/compliance/data-center/>.

Annual penetration tests conducted by a 3rd party specialist security testing company. The purpose of the test is to verify whether robust security mechanisms are in place to prevent unauthorised users from accessing data and infrastructure. This penetration test includes:

- Identification of potential vulnerabilities occurring in the application and defining possible attack scenarios conducted with techniques typical for attacks on web applications;
- Simulated attacks from the perspective of an anonymous and standard user;
- Testing API endpoints from the perspective of an anonymous and standard user, including mechanisms such as user authentication, access control, and data validation;
- Security assessment of our infrastructure against the latest industry standard AWS CIS Foundations Benchmark.

The AWS Compliance Program provides further assurance and understanding of the robust controls in place to maintain security and compliance in the cloud. AWS regularly achieves third-party validation for thousands of global compliance requirements that are continuously monitored to meet security and compliance standards for the most sensitive data and privacy requirements. AWS supports more security standards and compliance certifications than any other offering, including PCI-DSS, HIPAA/HITECH, FedRAMP, GDPR, FIPS 140-2, and NIST 800-171, helping satisfy compliance requirements for virtually every regulatory agency around the globe. More information can be found by visiting <https://aws.amazon.com/compliance/programs/>.

**Peek Platform Data Security Controls:**

**Peek Servers:**

Peek servers hosted in a Virtual Private Cloud (VPC) utilising the Amazon Web Services (AWS) Cloud. Each Peek powered programme is hosted on it’s own dedicated server and a VPC that will reside in the UK/EU ensuring all of the data privacy safeguards as governed under the GDPR.

Server OS is Amazon Linux ustlising AWS AMIS to provide base images for our system drives and enhances security by focusing on two main security goals, limiting access and reducing software vulnerabilities. Security updates are applied automatically to test once a week and then rolled out a week later automatically to other environments

**Docker:**

Peek server software runs in Docker containers. Docker shields application software from variations in platform and co-hosted software. It ensures that development, test and production environments run the same context as one another to ensure consistent, predictable behaviour. Peek servers also use docker swarm mode to achieve failsafe reliability and replication of Mongo databases.

**Databases:**

Server data is stored in Mongo databases, a fast, scalable, json document database. Peek infrastructure uses a Mongo replica set across two hosts. There are two replicas each holding a full copy of the data and one arbiter. The arbiter is only used for the election of a new master if one of the nodes was to become unavailable. The Mongo database and journal are held on AWS Secure EBS volumes. This provides 256-bit AES encrypted using a key managed under the Amazon Key Management Service.

Amazon Key Management Service, allows us to create and manage cryptographic keys and securely control their use across a wide range of AWS services and within our applications. AWS KMS is a secure and resilient service that uses hardware security modules that have been validated under FIPS 140-2 to protect the encryption keys. AWS KMS also integrates with AWS CloudTrail providing us with secure logs of all key usage. Backups on S3 are also encrypted using keys managed by AWS Key Management Service.

**Logging and Monitoring:**

Peek Server and Mongo Server logs and uploaded to AWS Cloudwatch for storage and monitoring. AWS Cloudwatch collects monitoring and operational data in the form of logs, metrics, and events and alerts us immediately of problems in any environment, both application and infrastructure.

**Network Security:**

AWS Security groups are used to provide firewall-like network access control and allow inbound traffic on HTTP and HTTPS ports. Outbound traffic is permitted on any port. The SSH traffic is restricted to subnets associated with devops engineers and the deployment servers. TLS 1.2 is used to secure traffic between device or browser and server.

Operational access to the AWS console is protected with AWS IAM MFA which uses 2-Factor Authentication and ensures that access to AWS is restricted to users with knowledge of password and possession of a specific approved mobile device. Automated access to the AWS API uses AWS Roles with restricted privileges needed for housekeeping, logging and alarm maintenance. No user use is made of Access Keys to eliminate the vulnerabilities of file-system-based credentials.

**Threat Detection:**

AWS Guard Duty is enabled, this provides a threat detection service that continuously monitors for malicious activity and unauthorised behaviour to protect access, workloads and data. The service utilises up-to-date threat intelligence feeds from AWS, CrowdStrike, and Proofpoint and continuously evolves through machine learning.

**Backups:**

An Image is maintained of the Server Host using AWS AMI to ensure continuous availability.

A snapshot of the encrypted data volume, containing database and journal, is taken four times daily. Snapshots are retained for two weeks. Access to the snapshots is strictly controlled. Old backups are automatically deleted after 90 days. Backups are stored on AWS S3 storage, also encrypted providing 256-bit AES encryption. The backups are stored across AWS multiple availability zones, this ensures that the data resides in multiple data centres separated geographically and stored in AWS secure data centres.

Additionally  a further backup is made off AWS. Off-AWS backups are replicated to Google Cloud daily via Google Transfer service to identically named buckets and files with a retention policy of 90 days.

**Data Centres:**

All data collected is securely stored in AWS data centres which are state of the art, utilising innovative architectural and engineering approaches.

**Disaster Recovery:**

A full disaster recovery test is performed at least annually to ensure servers, applications and databases can be fully recovered within 24 hours.

**Export/data sharing for analysis**

At the analysis stage pseudo-anonymised data will be exported in an encrypted zip file CSV file to LSHTM researchers to perform statistical testing. The zip file will be saved on the protected LSHTM server and only named project staff will be given access. Passwords will be sent separately. We will only ever export the minimum data required for the analyses.

Labelling conventions

1. Keep file names short, meaningful and easily understandable to others.
2. Order the elements in a file name in the most appropriate way to retrieve the record.
3. Avoid unnecessary repetition and redundancy in file names and paths
4. Avoid obscure abbreviations and acronyms. Use agreed University abbreviations and codes where relevant.
5. Avoid vague, unhelpful terms such as “miscellaneous” or “general” or “my files”
6. Use capital letters to delimit words, as the preferred option, although underscores (_) or hyphens (-) may add clarity, they make the file name longer.
7. For numbers 0-9, always use a minimum of two digit numbers to ensure correct numerical order (e.g. 01, 02, 03 etc.)
8. Dates should always follow same format: YYYYMMDD e.g. 20170425
9. When including a personal name give the family name first followed by initials, with no comma in between e.g. SmithAB
10. Avoid using common words such as ‘draft’ or ‘letter’ at the start of file names unless doing so will make it easier to retrieve the record.
11. Use alphanumeric characters i.e. letters (A-Z) and numbers (0-9). Avoid using invalid characters in file names such as *? \ / : # % ~ { }
12. The file names of records relating to recurring events should include the date and a description of the event, except where the inclusion of these elements would be incompatible with rule 3.
13. The version number of a record should be indicated in its file name by the inclusion of ‘V’ followed by the version number (e.g. V01, V03 etc.). However versioning is enabled automatically in systems such as Office 365 and One Drive for Business, making it unnecessary to duplicate this information in the file name itself. e.g. 2021-11-19_Topic_Filename-variable01

Measures to keep data safe and secure

| Only anonymised data will be used - personal, sensitive, or otherwise confidential data is not needed for the research |  | Store personal details in a separate secure location & link it via an identifier | Not required | Delete personal & confidential details at earliest opportunity (specify when below) | X |
| --- | --- | --- | --- | --- | --- |
| Use digital storage that require a username/password or other security feature | X | Physical security (such as locked cabinet or room) | X | Protect portable devices using security features, e.g. biometric |  |
| Encrypt storage devices | X | Encrypt during transfer | X | Avoid cloud services located outside EU | X |
| Take ‘Information Security Awareness training’ | X | Ensure backups are also held securely | X |  |  |
| Notes: | The aggregated Peek data that is shared with LSHTM project staff will not contain any names, however the data being shared may still permit the identification of individuals depending on the domains being shared and may therefore constitute pseudo-anonymised data.    We also note that there is not adequate shared secure storage space at LSHTM. We will have to use our personal H drives which is suboptimal for joint working and version control. | | | | |

**ARCHIVING & SHARING**

- All data will be stored for 10 years.
- Internal confidential files will be retained on Peek’s secure servers.
- LSHTM analyses will be saved on encrypted and password-protected files on LSHTM SharePoint, with access restricted to the project team. Once the project is complete these files will be moved to a secure server.
- Data presented in publications (anonymised aggregate mean attendance rates for each SES subgroup) will be published on GitHub.

When will the resources be made available?

| During the research life |  | At the same time as findings are published in an academic journal | x | A set time after research end, e.g. 12 months. Specify below |  |
| --- | --- | --- | --- | --- | --- |
| Resources already available (provide details below) |  | On completion of my thesis |  | Other (provide details below) |  |
| Further information / Other | | | | | |
|  | | | | | |

How will you make other researchers aware that the resources exist?

| Publish a metadata record describing the resources in a repository or other catalogue |  | Obtain a Digital Object Identifier (DOI) or other permanent ID |  |
| --- | --- | --- | --- |
| Cite resources in future research papers, e.g. in the data access statement or reference list | x | Cite resources in project reports | x |
| Publish a description for the project website |  | Write and publish a Data Paper |  |
| Add resources to a list of your academic outputs | x |  |  |
| Other measures / Further details | | | |
|  | | | |

What steps will you take to ensure resources are easy to analyse and use in future research?

| Prepare a codebook or other documentation that provides an accurate description of content |  | Store resources in open file formats such as CSV, Rich Text, etc. See <https://www.ukdataservice.ac.uk/manage-data/format/recommended-formats> | x |
| --- | --- | --- | --- |
| Write a user guide that provides a high-level overview of research |  | Apply a standard licence that allows a broad range of uses (e.g. Creative Commons, Open Data Commons) |  |
| Designate a corresponding author / data custodian who will handle data-related questions | x | Use domain-specific standards that make it easy to import and analyse data |  |
| Other / Further information | | | |
|  | | | |

If resources can be made available, but not openly, what conditions on access/use must be met?

E.g. data can be used for specific types of research only. Leave blank if not applicable.

| **Requirement:** | **To be addressed by:** |
| --- | --- |
| In line with the UK concordat on open research data (2016), anonymised data from this trial will be made available to bona fide research groups (evidenced via CVs and the involvement of a qualified statistician), and in line with the trial’s publicly available data sharing policy, following review and approval from the trial’s data monitoring committee. No reasonable request will be turned down, and the appropriate data will be made available within 1-month of receiving the request.    There may be multiple levels of permission required in-country before data can be shared, including:  National ministry of health approval  +- Local implementation partner approval | The PI will forward requests for data to the in-country leads in order to seek the relevant permissions. We will respond to any boa fide request within 28 days. |

**RESOURCING**

Primary data management challenges

With respect to costs of resources, we have adequate funding within the Wellcome project grant. The data is collected through active live Peek powered programmes where funding and resources is already provided for data collection and data security.
